# Clinical and Bacterial Characteristics Associated with Glove and Gown Contamination by Carbapenem-Resistant Klebsiella pneumoniae in the Health Care Setting

**DOI:** 10.1128/spectrum.01775-23

**Published:** 2023-06-08

**Authors:** Tracy H. Hazen, Timileyin Adediran, Stephanie Hitchcock, Lyndsay M. O’Hara, Lisa Pineles, Jane M. Michalski, J. Kristie Johnson, M. Hong Nguyen, David P. Calfee, Loren G. Miller, Anthony D. Harris, David A. Rasko

**Affiliations:** a Institute for Genome Sciences, University of Maryland School of Medicine, Baltimore, Maryland, USA; b Department of Microbiology and Immunology, University of Maryland School of Medicine, Baltimore, Maryland, USA; c Department of Epidemiology and Public Health, University of Maryland School of Medicine, Baltimore, Maryland, USA; d Department of Medicine, University of Pittsburgh, Pittsburgh, Pennsylvania, USA; e Division of Infectious Diseases, Weill Cornell Medicine, New York, New York, USA; f Lundquist Institute at Harbor-UCLA Medical Center, Torrance, California, USA; Universitat Greifswald

**Keywords:** *Klebsiella pneumoniae*, genomics, health care provider, transmission

## Abstract

Carbapenem-resistant Klebsiella pneumoniae (CRKp) is a pathogen of significant concern to public health, as it has become increasingly associated with difficult-to-treat community-acquired and hospital-associated infections. Transmission of K. pneumoniae between patients through interactions with shared health care personnel (HCP) has been described as a source of infection in health care settings. However, it is not known whether specific lineages or isolates of K. pneumoniae are associated with increased transmission. Thus, we used whole-genome sequencing to analyze the genetic diversity of 166 carbapenem-resistant K. pneumoniae isolates from five U.S. hospitals in four states as part of a multicenter study examining risk factors for glove and gown contamination by carbapenem-resistant *Enterobacterales* (CRE). The CRKp isolates exhibited considerable genomic diversity with 58 multilocus sequence types (STs), including four newly designated STs. ST258 was the most prevalent ST, representing 31% (52/166) of the CRKp isolates, but was similarly prevalent among patients who had high, intermediate, and low CRKp transmission. Increased transmission was associated with clinical characteristics including a nasogastric (NG) tube or an endotracheal tube or tracheostomy (ETT/Trach). Overall, our findings provide important insight into the diversity of CRKp associated with transmission from patients to the gloves and gowns of HCP. These findings suggest that certain clinical characteristics and the presence of CRKp in the respiratory tract, rather than specific lineages or genetic content, are more often associated with increased transmission of CRKp from patients to HCP.

**IMPORTANCE** Carbapenem-resistant Klebsiella pneumoniae (CRKp) is a significant public health concern that has contributed to the spread of carbapenem resistance and has been linked to high morbidity and mortality. Transmission of K. pneumoniae among patients through interactions with shared health care personnel (HCP) has been described as a source of infection in health care settings; however, it remains unknown whether particular bacterial characteristics are associated with increased CRKp transmission. Using comparative genomics, we demonstrate that CRKp isolates associated with high or intermediate transmission exhibit considerable genomic diversity, and there were no K. pneumoniae lineages or genes that were universally predictive of increased transmission. Our findings suggest that certain clinical characteristics and the presence of CRKp, rather than specific lineages or genetic content of CRKp, are more often associated with increased transmission of CRKp from patients to HCP.

## INTRODUCTION

Carbapenem-resistant *Enterobacterales* (CRE) are a significant concern in health care settings (1) and have been identified by the Centers for Disease Control and Prevention as one of five urgent threats to public health in the United States (2). The CRE have also been designated by the World Health Organization as a pathogen of critical priority for additional research and the development of new antibiotics for their treatment (3). In particular, the carbapenem-resistant Klebsiella pneumoniae (CRKp) strains are of concern as they have contributed to the spread of carbapenem resistance and have been linked to high mortality of critically ill patients (4–8). Carbapenem resistance among K. pneumoniae isolates has most often been attributed to the K. pneumoniae carbapenemase (KPC), and successful lineages of K. pneumoniae, such as sequence type 258 (ST258), have had a major role in the spread of *bla*_KPC_ genes (7, 9–12).

Patient-to-patient transmission of K. pneumoniae has also been identified as a significant source of infection in health care settings (13, 14). Previous studies have demonstrated that the gloves and gowns of health care personnel (HCP) frequently become contaminated by antibiotic-resistant bacteria, including methicillin-resistant Staphylococcus aureus (MRSA) and vancomycin-resistant enterococci (VRE), after caring for patients with these bacteria (15–17). Thus, glove and gown contamination has been examined as a surrogate measure for examining factors that contribute to patient-to-patient transmission (18). Glove and gown contamination is an appropriate surrogate measure for transmission in the health care setting for the following reasons: (i) it is an intermediate step in the transmission event from a colonized patient to an uncolonized patient; (ii) it represents transmission to the health care worker from the patient during the health care worker-patient interaction; (iii) it denotes the bacteria that could be “transported” or “spread” to the next patient if hand hygiene is not performed or is suboptimal; and (iv) it occurs frequently, allowing for adequately powered, cost-effective studies. K. pneumoniae was also identified on the gloves and gowns of HCP, although there were no differences in the frequency of contamination by KPC-producing compared to non-KPC-producing K. pneumoniae (19). Types of HCP-patient interactions, increased bacterial burden, positive clinical culture, and being in an intensive care unit (ICU) have been linked to increased CRE transmission (18). However, much remains unknown about whether there are characteristics of certain K. pneumoniae isolates that render them more likely to contaminate the gloves and gowns of HCP.

Whole-genome sequencing has been used to investigate the diversity of K. pneumoniae isolates exhibiting antibiotic resistance, hypervirulence, and association with outbreaks (10, 13, 20–23). However, it is not known whether certain lineages or genes are more often associated with patient-to-patient transmission of K. pneumoniae. Thus, our study aims to gain a better understanding of whether certain bacterial characteristics of K. pneumoniae, such as particular lineages or genetic content, are more often associated with HCP glove or gown contamination. The identification of specific bacterial characteristics among CRKp isolates associated with increased transmission could be used by HCP to inform patient management plans and interventions to prevent the spread of this significant pathogen in the hospital. The objective of our study was to characterize the genomic diversity of CRKp associated with glove and gown contamination in the hospital setting and determine whether characteristics of CRKp isolates are associated with differences in their observed frequency of transmission from patients to HCP. The 166 CRKp isolates analyzed in our study were obtained from clinical or surveillance samples from five hospitals in four states and were associated with high, intermediate, or low transmission based on the frequency of HCP glove or gown contamination (18). We used comparative genomics to examine the genetic diversity of these CRKp isolates and investigate whether patient and/or bacterial characteristics are associated with differences in the frequency of glove and gown contamination by CRKp.

## RESULTS

### Sources of the CRKp isolates and their association with transmission.

A total of 166 CRKp patient isolates were analyzed in this study, among which 8% (13/166) were associated with high transmission, 35% (58/166) with intermediate transmission, and 57% (95/166) with low transmission. Transmission was determined by the frequency at which K. pneumoniae was cultured from the gloves and gowns of HCP when HCP had completed their patient care interactions (24). The CRKp isolates were from five hospitals that included two located in Maryland (MD) and one each in California (CA), New York (NY), and Pennsylvania (PA). More than half (63%, 104/166) of the isolates were obtained from the two hospitals in Maryland (MD-A and MD-B) (Table 1). The CRKp were isolated between 2016 and 2019 and included similar numbers of isolates from ICU (54%, 89/166) and non-ICU (46%, 77/166) patients. A greater number of the CRKp isolates were from clinical samples (77%, 128/166), which included blood, sputum, wound, urine, and other cultures, compared to 23% (38/166) of the isolates being from perirectal cultures obtained for routine infection control surveillance purposes.

**TABLE 1:** Transmission and sample

Sample subgroup	No. of CRKp isolates (%)^*a*^		*P* value^*c*^
	High and intermediate transmission	Low transmission	
All CRKp isolates	71	95	
Hospital^*b*^	High and intermediate	Low	0.33
MD-A	28 (39)	49 (52)	0.12
MD-B	11 (15)	16 (17)	0.82
NY	19 (27)	14 (15)	0.05
CA	7 (10)	7 (7)	0.57
PA	6 (8)	9 (9)	0.82
Yr	High and intermediate	Low	0.88
2016	13 (18)	20 (21)	0.66
2017	28 (39)	34 (36)	0.63
2018	20 (28)	30 (32)	0.64
2019	10 (14)	11 (12)	0.63
Source	High and intermediate	Low	0.01
ICU	46 (65)	43 (45)	0.01
Non-ICU	25 (35)	52 (55)	0.01
Sample type	High and intermediate	Low	0.4
Clinical	57 (80)	71 (75)	0.40
Sputum	19 (27)	7 (7)	0.001
Wound	8 (11)	6 (6)	0.26
Blood	4 (6)	13 (14)	0.09
Urine	18 (25)	25 (26)	0.89
Other	8 (11)	20 (21)	0.09
Surveillance (perirectal swab)	14 (20)	24 (25)	0.40

a High, intermediate, and low transmission was determined by the frequency at which the CRKp isolate was cultured from the gloves and gowns of health care personnel. Each percentage was calculated relative to the total number of high- and intermediate-transmission and low-transmission isolates (top row).

b Maryland hospital A (MD-A), Maryland hospital B (MD-B), California (CA), Pennsylvania (PA), and New York (NY).

c *P* values were calculated using the chi-square test.

There were similar proportions of CRKp associated with high, intermediate, and low transmission from each of the hospitals, as well as from each year of the study (Table 1). ICU patients had a greater number of CRKp isolates associated with high and intermediate transmission (65%, 46/71) than with low transmission (45%, 43/95) (*P* = 0.01), while the opposite was true for non-ICU patients. We also examined CRKp transmission relative to the sample types from which CRKp isolates were obtained. There were similar proportions of the high- and intermediate-transmission CRKp isolates and of the low-transmission CRKp isolates from both clinical and surveillance samples. The CRKp isolates from patients who had high and intermediate transmission were more often from sputum (27%; 19/71) than were CRKp isolates associated with low transmission (7%; 7/95) (*P* = 0.001). Among the 38 CRKp isolates from perirectal surveillance swabs, 37% (14/38) were from patients who had high or intermediate CRKp transmission compared to 63% (24/38) from patients who had low CRKp transmission (*P* = 0.038).

### Clinical characteristics associated with increased CRKp transmission.

We also examined whether certain clinical characteristics were more prevalent among patients with high or intermediate CRKp transmission than among those with low transmission. Notably, 61% (43/71) of the patients who had high or intermediate CRKp transmission had a nasogastric (NG) tube, compared to 29% (28/95) of the patients who had low CRKp transmission (*P* < 0.0001) (Table 2). Also, 62% (44/71) of the CRKp isolates associated with high and intermediate transmission, compared to 43% (41/95) of the CRKp isolates associated with low transmission, were from patients who had an endotracheal tube or tracheostomy (ETT/Trach) (*P* = 0.02).

**TABLE 2:** Clinical characteristics of patients who had the CRKp isolates

Clinical characteristic	No. of CRKp isolates (%)^*a*^		*P* value^*b*^
	High and intermediate transmission (*n* = 71)	Low transmission (*n* = 95)	
ETT/Trach	44 (62)	41 (43)	0.02
Wound	59 (83)	66 (69)	0.04
Foley catheter	33 (46)	38 (40)	0.41
CVC/PICC^*c*^	44 (62)	55 (58)	0.6
Chest tube	3 (4)	6 (6)	0.56
Surgical drain	20 (28)	24 (25)	0.67
Diarrhea	24 (34)	29 (31)	0.65
Rectal tube	14 (20)	11 (12)	0.15
NG tube	43 (61)	28 (29)	<0.0001

a Each percentage was calculated relative to the total number of high- and intermediate-transmission and low-transmission isolates. Across all clinical characteristics, the *P* value was 0.53.

b *P* values were calculated using the chi-square test.

c CVC, central venous catheter; PICC, peripherally inserted central catheter.

The odds of glove or gown contamination with CRKp differed by HCP type (Table 3). Respiratory therapists had the greatest odds of glove or gown contamination, with 20% of all interactions resulting in CRKp contamination (adjusted odds ratio [aOR], 5.52; 95% confidence interval [CI], 1.67 to 18.18). The second highest odds of contamination were observed among occupational and physical therapists (aOR, 4.60; CI, 1.16 to 18.32) followed by nurses (aOR, 4.16; CI, 1.51 to 11.47), compared to HCP in the “other” category (e.g., social workers, nutritionists, etc.). As expected, HCP interactions that included only contact with the environment were associated with decreased odds of transmission of CRKp to the gloves or gown (aOR, 0.53; CI, 0.28 to 1.00) (see Table S1 in the supplemental material) compared to those of interactions that included contact with the patient.

**TABLE 3:** Association between health care personnel type and contamination of gloves or gowns with CRKp^*b*^

Type of healthcare personnel (*n* = 1,658)	No. of observations (%)	Interactions resulting in contamination (%)	aOR (95% CI)	*P* value
Respiratory therapist	85 (5.1)	20	5.52 (1.67–18.18)	0.005
Occupational/physical therapist	59 (3.6)	11.9	4.60 (1.16–18.32)	0.03
Nurse	889 (53.6)	13.7	4.16 (1.51–11.47)	0.006
Patient care technician	177 (10.7)	10.2	3.31 (1.06–10.33)	0.04
Environmental services	96 (5.8)	9.4	3.28 (0.94–11.42)	0.06
Medical doctor/nurse practitioner	224 (13.5)	7.6	1.63 (0.52–5.12)	0.4
Other^*a*^	128 (7.7)	3.9	Ref	Ref

a Includes social workers, nutritionists, researchers, etc.

b Values adjusted for culture source and ICU status. Abbreviations: CI, confidence interval; OR, odds ratio; Ref, reference.

### Identification of the CRKp isolates in diverse K. pneumoniae lineages.

The species designation of all 166 CRKp isolates analyzed in this study was verified by Kleborate (25) analysis of the genomes, which identified all of the CRKp isolates as K. pneumoniae. Phylogenomic analysis of the CRKp isolates along with representative genomes of the species belonging to the K. pneumoniae species complex (KpSC) (25) also demonstrated the relatedness of all 166 CRKp isolates with K. pneumoniae compared to representative genomes of other KpSC members (Fig. S1). There were 58 multilocus sequence types (MLSTs) identified among the CRKp isolates, including four newly designated STs (ST6005, ST6006, ST6007, and ST6008) and 39 STs represented by a single isolate (Data Set S1). The CRKp isolates associated with high or intermediate transmission (*n* = 71) had 26 STs while the isolates associated with low transmission (*n* = 95) had 43 STs. ST258 was the most prevalent ST, representing 31% (52/166) of the CRKp isolates, and was similarly prevalent among patients who exhibited high (31%, 4/13), intermediate (38%, 22/58), and low (27%, 26/95) CRKp transmission. None of the STs were significantly more prevalent among patients with high, intermediate, or low CRKp transmission (Data Set S2).

The CRKp isolates were also assigned to previously described K. pneumoniae clonal groups (CGs) (26, 27). Multidrug-resistant (MDR) K. pneumoniae isolates have been identified with many different STs (21); however, the STs of particular concern are those that group into the eight globally distributed MDR CGs (CG15, CG20, CG29, CG37, CG147, CG101, CG258, and CG307) or the CGs that have been linked to hypervirulence (CG23, CG25, CG65, CG66, CG86, and CG380) (26, 27). There were 55% (92/166) of the CRKp isolates assigned to five of the eight global MDR CGs (CG15, CG20, CG37, CG258, and CG307) (Data Set S2). There were 77% (10/13) of the high-transmission, 62% (36/58) of the intermediate-transmission, and 48% (46/95) of the low-transmission isolates assigned to global MDR CGs. The most prevalent MDR clone among the CRKp isolates was CG258 with 38% (63/166) of the isolates. CRKp isolates of CG258 were significantly more prevalent among non-ICU patients (*P* = 0.013); however, this is likely related to 100% (8/8) of the ST11 isolates of this clone being from non-ICU patients whereas there were similar numbers of ST258 isolates from ICU (*n* = 23) and non-ICU (*n* = 29) patients. None of the CRKp isolates in this study were identified in clones linked to hypervirulence.

**FIG 1:**
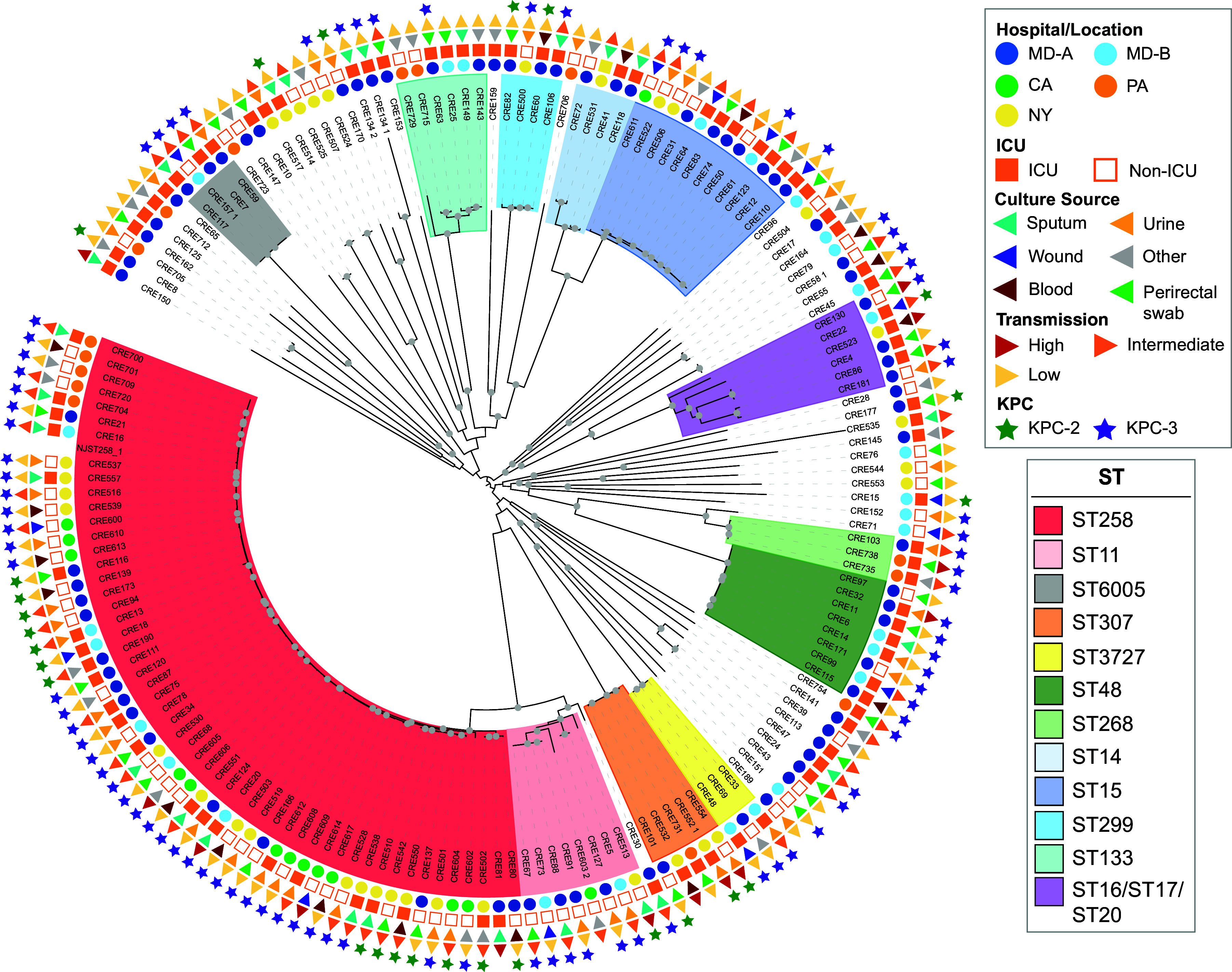
Phylogenomic analysis of CRKp isolates associated with high, intermediate, or low transmission from patients to the gloves and gowns of health care personnel. A maximum-likelihood phylogeny was inferred based on SNP sites identified among 166 CRKp isolates relative to K. pneumoniae NJST258_1 (GCA_000598005.1). Colors indicate the clades with three or more CRKp genomes of the same sequence type (ST). Symbols on the exterior of the phylogeny indicate the hospital/location, ICU or non-ICU, sample type from which each CRKp isolate was cultured, transmission status, and the *bla*_KPC_ allele identified in each CRKp isolate. The phylogeny was midpoint rooted, and UFBoot values of ≥95% are indicated by a gray circle.

Phylogenomic analysis further demonstrated the considerable diversity among the CRKp isolates in our study (Fig. 1). CRKp isolates associated with high, intermediate, and low transmission were distributed throughout the phylogeny, suggesting there was no clear association of any lineage of K. pneumoniae with increased transmission. The ST133 CRKp isolates (*n* = 6) were all associated with low transmission and were from perirectal surveillance swabs or other sources. Several lineages were made up of CRKp isolates from the same hospital or state, such as the ST48, ST6005, and ST3727 lineages that contained only CRKp isolates from hospitals in Maryland (MD-A and MD-B).

### Genomic comparisons of the ST258 CRKp isolates associated with different levels of transmission.

The ST258 CRKp isolates were further examined for genetic content associated with differences in transmission, as this was the most prevalent lineage identified. We examined whether certain genes were more or less prevalent among the ST258 CRKp isolates associated with high and intermediate compared to low transmission. This analysis grouped the predicted genes of the ST258 CRKp isolates into 8,632 total gene clusters that had ≥90% nucleotide identity and ≥90% alignment length. There were no ST258 CRKp genes that were significantly associated with differences in transmission or with ST258 CRKp isolates from the ICU versus non-ICU patients or from clinical versus surveillance samples. Genes that had a significantly different prevalence by hospital included three genes exclusively identified among 50% (8/16) of the isolates from MD-A and absent among the ST258 isolates from all other hospitals (Data Set S2). Also, there were 399 genes that were more prevalent among the ST258 isolates from PA (*n* = 5) than among those from the other hospitals or vice versa. Many of these genes have predicted functions involved in heavy metal resistance or conjugative transfer and are associated with mobile elements in the chromosome or on plasmids.

### K. pneumoniae virulence factors and CRKp transmission.

**FIG 2:**
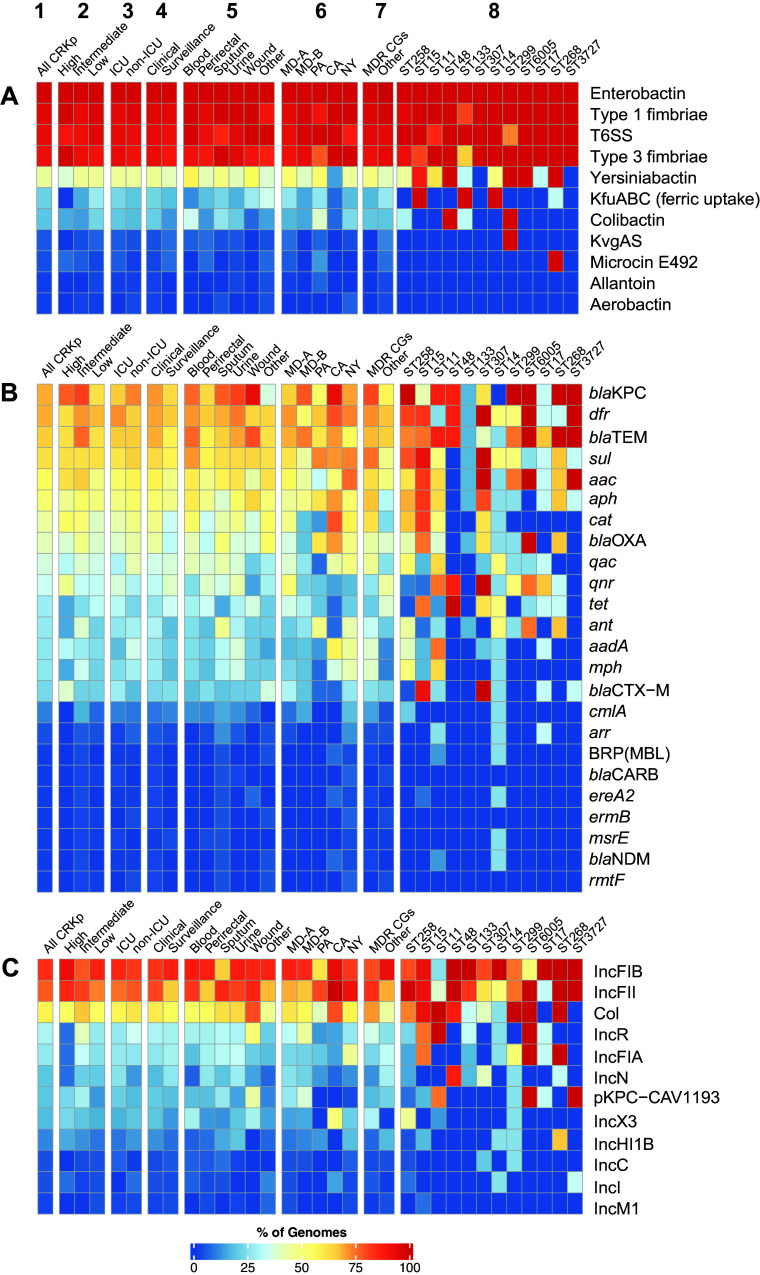
Prevalence of virulence factors, acquired antibiotic resistance genes, and plasmids among the CRKp isolates. The percentages of CRKp genomes in each category (1 to 8) that contain the previously described K. pneumoniae virulence factors (A), acquired antibiotic resistance genes (B), or plasmids represented by known incompatibility types (Inc) (C). The genomes were sorted into different categories for comparison (1, all 166 CRKp isolates; 2, transmission; 3, ICU versus non-ICU; 4, clinical versus surveillance; 5, sample type; 6, hospital; 7, MDR CGs versus other CRKp isolates; 8, MLSTs). Each row represents a different known virulence factor, resistance gene, or plasmid. Only the virulence factors, antibiotic resistance genes, and plasmids identified in two or more CRKp isolates are included in the heat maps. Features detected in single CRKp genomes are included in the supplemental material.

Detection of known K. pneumoniae virulence genes among the CRKp isolates demonstrated that none of the examined virulence factors were significantly more associated with high- or intermediate-transmission isolates than with low-transmission isolates (Fig. 2A; Data Set S2). Also, only four of the CRKp isolates contained the genes associated with hypervirulence of K. pneumoniae, such as the genes for allantoin metabolism (*all*) and aerobactin synthesis (*iuc*) (6) (Data Set S1 and Fig. S2). Only one of these CRKp isolates was from a patient who had intermediate transmission, while the other three were associated with low transmission. The genes *kfuABC*, which are involved in ferric uptake, were more frequent among the low-transmission CRKp isolates than the high- or intermediate-transmission isolates (*P* = 0.02) (Data Set S2).

Another genomic region associated with K. pneumoniae virulence is an integrative conjugative element (ICE*Kp*), which encodes the siderophore yersiniabactin involved in iron acquisition, and in some cases also colibactin, which is toxic toward eukaryotic cells (28). The yersiniabactin locus (*ybt*) was identified in 46% (76/166) of the CRKp isolates, including 49% (31/63) of the CG258 isolates, and was similarly distributed among the high (38%, 5/13)-, intermediate (47%, 27/58)-, and low (46%, 44/95)-transmission isolates (Data Set S2). The colibactin locus (*clb*) was identified in only 19% (32/166) of the CRKp isolates, all of which also contained the yersiniabactin locus and ICE*Kp*10. Colibactin also exhibited similar prevalences among CRKp isolates associated with high (15%, 2/13), intermediate (14%, 8/58), and low (23%, 22/95) transmission.

There were 9 different O serotypes predicted among the CRKp isolates analyzed. The two most common O serotypes were O1/O2v1 (30%, 50/166) and O1/O2v2 (49%, 82/166) (Data Set S2). O1/O2v2 was more prevalent among the CRKp isolates associated with high and intermediate transmission (62%, 44/71) than among those associated with low transmission (40%, 38/95) (*P* = 0.007). Also, O1/O2v2 was more prevalent among the CRKp isolates from non-ICU patients (62%, 48/77) than among those from ICU patients (38%, 34/89) (*P* = 0.002), whereas O1/O2v1 was more prevalent among the CRKp isolates from ICU patients (38%, 34/89) than among those from the non-ICU patients (21%, 16/77) (*P* = 0.017). As previously described, different O serotypes were associated with certain STs (29, 30) and were more prevalent at the different hospitals. There were 45 unique capsule types (KL) identified among the CRKp isolates (Data Set S2). The most prevalent KL was KL107, which was identified among 25% (41/166) of the CRKp isolates, all of which were in CG258. KL74 was identified among four of the high- or intermediate-transmission isolates and none of the low-transmission isolates (*P* = 0.03); however, there were few of these isolates, and further investigation would be required to determine whether isolates with this KL are more often associated with transmission.

### Plasmids and acquired antibiotic resistance genes.

The most prevalent carbapenem resistance mechanism identified among the CRKp isolates was KPCs (Fig. 2B). A *bla*_KPC_ gene was identified in 69% (115/166) of the CRKp isolates, with 53% (88/166) containing *bla*_KPC-3_, 16% (26/166) containing *bla*_KPC-2_, and one genome with a *bla*_KPC_ gene that exhibited 99% nucleotide identity and contained a 6-bp deletion relative to *bla*_KPC-79_ (NG_071205.1). A *bla*_NDM_ gene was identified in two of the CRKp isolates that did not contain a *bla*_KPC_ gene (Data Set S1). The 49 CRKp isolates that did not contain *bla*_KPC_ or *bla*_NDM_ genes may have carbapenem resistance conferred by other mechanisms such as increased expression of chromosomally encoded efflux pumps or AmpC-type β-lactamases or by the disruption of outer membrane porins such as OmpK35 and/or OmpK36 (27, 31). Also, *bla*_OXA-232_, which is known to exhibit carbapenemase activity (32), was identified in three of the CRKp isolates that did not contain *bla*_KPC_ (Data Set S1). Additionally, 43% (22/51) of the CRKp isolates that did not have *bla*_KPC_ did contain an extended-spectrum β-lactamase (ESBL) (*bla*_CTX-M-15_), which when coupled with the disruption of outer membrane porins can confer carbapenem resistance (33, 34).

Additional acquired resistance genes that confer resistance to aminoglycosides, ampicillin, fluoroquinolones, chloramphenicol, and sulfonamide were identified among the CRKp isolates. The most prevalent acquired antibiotic resistance genes identified encode aminoglycoside phosphotransferases [*aph(6)-Id*, *aph(3″)-Ib*, *aph(3′)-Ia*, *aph(3′)-VI*, and *aph(4)-Ia*], which were identified in 51% (84/166) of the CRKp isolates. Also, sulfonamide resistance genes (*sul1*, *sul2*, and *sul3*) were detected in 62% (103/166) of the isolates, *bla*_TEM_ was identified in 66% (110/166) of the CRKp isolates (Fig. 2B; Data Set S2), and *bla*_OXA_ was identified in 41% (68/166) of the CRKp isolates. Several acquired antibiotic resistance genes, such as *bla*_KPC_, were more prevalent among the high- and intermediate-transmission isolates (80%, 57/71) than among the low-transmission isolates (61%, 58/95) (*P* = 0.01) (Data Set S2). There were no antibiotic resistance genes associated with clinical isolates more than with the surveillance isolates; however, select antibiotic resistance genes were more prevalent among CRKp isolates from certain hospitals, such as *aac(6′)-Ib-cr5* and *qnrS1*, which were identified among a greater number of the CRKp isolates from MD-A than the other hospitals (*P* < 0.005) (Data Set S2). Also, acquired resistance genes including the *bla*_KPC_ genes, *catI* encoding chloramphenicol resistance, *sul1* and *sul3* encoding sulfonamide resistance, and genes for aminoglycoside resistance [*aadA2*, *aac(3)-IV*, *aph(3′)-Ia*, and *aph(4)-Ia*] were more prevalent among the CRKp isolates in the global MDR CGs (CG15, CG20, CG37, CG258, and CG307 [27]) than among the other CRKp isolates (*P* < 0.005) (Data Set S2). Clustering and cooccurrence analyses further demonstrated associations of acquired antibiotic resistance genes with the different hospitals as well as with select lineages of K. pneumoniae represented by STs or CGs (Fig. S3).

There were 39 previously described plasmid types identified among the CRKp isolates. All isolates contained at least one plasmid type and up to nine different plasmid types per isolate (Fig. S4). The IncFII_K_ and IncFIB_K_ plasmids were the most prevalent among the CRKp isolates in our study (Fig. 2C). This finding was anticipated, as the IncFII_K_ plasmids have been linked in previous studies to the spread of *bla*_KPC_ genes and are prevalent among ST258 isolates (7). pKPC-CAV1193 (CP013325) (35) is a 49-kb plasmid that contains *bla*_KPC-2_ and *bla*_TEM-1_ and was more prevalent among the high- and intermediate-transmission isolates (28%; 20/71) than among the low-transmission isolates (13%; 12/95) (*P* = 0.01) (Data Set S2 and Fig. S5). This plasmid was also more prevalent at the Maryland hospitals and was identified in only one CRKp isolate from outside Maryland. The 32 CRKp isolates carrying pKPC-CAV1193 (CP013325) had 17 different STs and were similarly distributed among ICU and non-ICU patients and clinical and surveillance samples, as well as a wide variety of sample types (perirectal, urine, sputum, etc.) (Fig. S5). This plasmid was more prevalent among non-ST258 CRKp isolates (*P* = 0.01), which included 75% of the ST11 (6/8, *P* = 0.01), 100% of the ST3727 (*n* = 3, *P* = 0.006), and 100% of the newly designated ST6005 (*n* = 4, *P* = 0.001) isolates. Further investigation is needed to determine whether this plasmid and encoded products are associated with increased transmission or whether it more likely represents a plasmid that has successfully spread among diverse CRKp isolates within a single hospital.

## DISCUSSION

Previous studies have described the frequency of and risk factors for glove and gown contamination, which is a surrogate measure for patient-to-patient transmission of antibiotic-resistant bacteria in hospitals (15–19). However, much remains unknown about the diversity and unique gene content of CRKp isolates associated with increased transmission from patients to the gloves and gowns of HCP. Using comparative genomics, we demonstrate that CRKp isolates associated with increased transmission do not belong to a single K. pneumoniae lineage but instead exhibit considerable genomic diversity. Comparison of the genetic content among the ST258 CRKp isolates, which were the most prevalent lineage of K. pneumoniae in the study but exhibited similar associations with high, intermediate, and low transmission, suggests that specific genes are not associated with increased transmission to gloves and gowns of HCP.

While there were more of the CRKp isolates from high and intermediate transmission in several lineages of K. pneumoniae, the number of isolates in many of these lineages was small and would require further investigation to associate specific lineages with increased transmission. For instance, more of the ST307 isolates were associated with high transmission, which is interesting as ST307 has become increasingly more prevalent in certain regions of the world, including the United States (20, 27, 36–38). Also, ST307 isolates have been described with a plasmid-encoded glycogen synthesis locus, which was proposed to facilitate survival under nutrient limitation in the environment and may provide an advantage in hospitals (36). However, the number of ST307 isolates included in our study was limited (*n* = 5), and additional studies would be required to further investigate the association of ST307 CRKp isolates with increased transmission. There were also lineages of K. pneumoniae that were more associated with different hospitals included in this study. This is consistent with previous studies that demonstrated regional association of certain K. pneumoniae lineages (27) and the emergence and persistence of subclades of ST258 in a single hospital over time (22).

More than half of the CRKp isolates in our study were identified in five of the eight most problematic MDR clones of K. pneumoniae, while none were identified in hypervirulent clones (27). As our study is focused on carbapenem-resistant isolates, this is to be expected as it has been previously demonstrated that K. pneumoniae MDR clones contain fewer virulence-associated genes, while hypervirulent clones contain fewer antibiotic resistance genes (21, 26, 27). The absence of genes associated with hypervirulence among all but one of the high- and intermediate-transmission isolates suggests these genes are not directly involved in increased transmission of CRKp. Additional virulence factors such as yersiniabactin and colibactin were present in multiple lineages among the CRKp isolates in our study; however, these loci and other virulence factors were not associated with the increased CRKp transmission. The O1/O2v2 serotype was associated with the high- and intermediate-transmission CRKp isolates, which included many of the CG258 isolates in our study, as expected based on the prior association of this serotype with CG258 (29, 30). Further investigation is necessary to determine whether the different O antigens and surface carbohydrates may provide an advantage to transmission.

As more than half of the CRKp isolates in our study were identified as belonging to MDR clones, the prevalence of acquired antibiotic resistance genes among these isolates was anticipated. Overall, we determined that plasmids and acquired antibiotic resistance genes identified among the CRKp isolates had similar prevalences among the high-, intermediate-, and low-transmission isolates. Also, select antibiotic resistance genes or plasmids were associated with certain K. pneumoniae lineages or hospitals, suggesting a clonal expansion of the isolates or plasmids carrying the genes in the different hospitals. There was one plasmid, pKPC-CAV1193 (35), that was more prevalent among the CRKp isolates associated with increased transmission. This plasmid was identified in CRKp isolates of different lineages, and all but one of the CRKp isolates carrying this plasmid were from the hospitals in Maryland, which suggests a proliferation of this plasmid among diverse CRKp isolates in the Maryland hospitals. Further investigation of this plasmid could provide insight into whether it confers an advantage for increased transmission.

In a previous study, we described increased CRE transmission from patients to HCP who have greater patient contact, such as respiratory therapists and nurses (18). Analysis of only the patients who had CRKp transmission similarly demonstrated that respiratory therapists and nurses were more likely to have glove and gown contamination by CRKp. However, we also determined that occupational therapists and patient care technicians had significant CRKp contamination, which was not significant when considering all CRE (18). There were also certain patient clinical characteristics associated with increased transmission. The patients who had high or intermediate CRKp transmission more often had an NG tube or ETT/Trach. K. pneumoniae can colonize or cause infection in a variety of body sites including the respiratory tract, wounds, urinary tract, and the gastrointestinal tract (6), and a previous study demonstrated relatedness among K. pneumoniae isolates from the same body site for isolates from blood, urinary, or respiratory samples (39). Further investigation of a greater number of K. pneumoniae isolates from the respiratory tract than from other body sites could provide insight into whether specific lineages of K. pneumoniae are better suited to colonization or infection of the respiratory tract.

In conclusion, our findings provide important insight into the genomic diversity of CRKp isolates from patients who had different frequencies of CRKp transmission to the gloves and gowns of HCP. While certain K. pneumoniae lineages contained a greater number of CRKp isolates associated with high and intermediate than with low transmission, and vice versa, the numbers of isolates for most lineages were small, and overall, there was no single K. pneumoniae lineage or specific genes that were universally predictive of increased transmission. Interactions with HCP, including nurses and respiratory therapists, who typically have greater patient contact, were associated with increased CRKp transmission. Also, certain clinical characteristics including an NG tube or an ETT/Trach, and a greater number of high- and intermediate-transmission CRKp isolates from sputum samples, suggest the presence of CRKp in the respiratory tract may be linked to increased transmission. Thus, our findings suggest that certain clinical characteristics and the presence of CRKp in the respiratory tract are more predictive of CRKp transmission than the presence of specific lineages or gene contents of CRKp.

## MATERIALS AND METHODS

### Study design.

The K. pneumoniae isolates analyzed in the current study were obtained as part of a multihospital study to investigate the transmission of carbapenem-resistant *Enterobacterales* (CRE) to the surrogate outcome of glove and gown contamination sampled after patient care; contamination of health care workers’ gloves and gowns after patient care is a surrogate for potential transmission to other patients. The study enrolled CRE-positive patients from two hospitals in Baltimore, MD, and one hospital each in Pittsburgh, PA, Torrance, CA, and New York, NY. Patients who were enrolled had a CRE-positive clinical or surveillance culture within 7 days of their enrollment. Research staff were stationed outside the patient room and cultured the gloves and gowns of 10 different HCP who interacted with each patient upon completion of patient care. The Institutional Review Board at each of the hospitals granted approval for waived consent of participants. Additional details of the study design are described elsewhere (18).

The transmission frequency of CRKp among the CRKp-positive patients enrolled in the study was determined by the number of times a CRKp isolate was cultured from the gloves and/or gowns of HCP who interacted with each patient (18). CRKp isolates associated with high transmission were detected in >50% of the glove and gown cultures, and intermediate-transmission CRKp isolates were in at least one but <50% of the cultures, while CRKp isolates associated with low transmission were not identified in any cultures. A potential limitation of our study is the possibility that multiple genomically distinct CRKp isolates were present in a single patient and the isolate analyzed using genomics in our study may not have been the isolate associated with the glove and gown contamination.

### Bacterial isolates.

Bacterial isolates were identified as K. pneumoniae using standard methodology at each hospital as previously described (24). Carbapenem resistance was determined by susceptibility testing to carbapenems and interpreted in accordance with CLSI guidelines (40).

### Genome sequencing and assembly.

K. pneumoniae isolates were grown overnight in lysogeny broth (LB) at 37°C. Total genomic DNA was extracted in 96-well format from 100 μL of sample using the MagAttract PowerMicrobiome DNA/RNA kit (Qiagen, Hilden, Germany) automated on a Hamilton Microlab Star robotic platform. Bead disruption was conducted on a TissueLyser II instrument (20 Hz for 20 min) in a 96-deep-well plate in the presence of lysis buffer and 200 μL phenol-chloroform. Genomic DNA was eluted in 90 μL water after magnetic bead cleanup and was quantified by Pico Green. The DNA was used to generate paired-end sequencing libraries using the KAPA HyperPrep kit and was sequenced 2 × on the Illumina NovaSeq platform. Prior to assembly, the raw sequencing reads were filtered to remove contaminating reads from the phiX sequencing control, using BBDuk of the BBTools software suite (https://sourceforge.net/projects/bbmap/). The raw reads were also trimmed to remove contaminating Illumina adaptor sequences and quality using Trimmomatic v.0.36 (41). The filtered reads were assembled using SPAdes v.3.14.1 (42), using the isolate assembly option for high-coverage bacterial isolate data.

### Species identification of the genomes.

The species-level taxonomic classification of each genome assembly was made using GTDB-Tk v2 (43). Species predictions were also made using Kleborate v.2.2.0 (25) run with default parameters. Only “strong” predictions were considered. The 166 CRKp genomes were phylogenomically compared to representative genomes of each closely related species that along with K. pneumoniae form the K. pneumoniae species complex (KpSC) (25) as described below.

### Phylogenomic analyses.

A single nucleotide polymorphism (SNP)-based approach was used to compare the whole-genome sequences of the 166 CRKp isolates (see Data Set S1 in the supplemental material) against representative genomes obtained from RefSeq for each species in the K. pneumoniae species complex (KpSC): K. pneumoniae (GCF_000016305.1), K. africana (GCF_900978845.1), Klebsiella quasipneumoniae subsp. *quasipneumoniae* (GCF_000751755.1), K. quasivariicola (GCF_002269255.1), K. variicola (GCF_000025465.1), Klebsiella quasipneumoniae subsp. *similipneumoniae* (GCF_000613225.1), and Klebsiella variicola subsp. *tropica* (GCF_900978435.1) (25) (Fig. S1). A second phylogenomic analysis was performed to examine the K. pneumoniae species-wide diversity of the 166 CRKp isolates, and SNP calling was performed relative to the K. pneumoniae ST258 reference genome NJST258-1 (GCA_000598005.1) (Fig. 1). SNPs were identified for each genome relative to the reference genome using the Northern Arizona SNP Pipeline (NASP) v.1.1.2 with default parameters (44). SNPs were filtered to remove sites in regions duplicated in the reference genome, sites with missing data, and monomorphic sites as described previously (44). A maximum-likelihood phylogeny was inferred for the SNP alignments using IQ-TREE v.1.6.12 with the GTR Gamma model run with ascertainment bias correction (GTR+G+ASC) (45). Bootstrap support was determined using ultrafast bootstrap approximation (UFBoot2) run with 1,000 replicates and the bnni option to reduce overestimating support (46). The phylogenies were midpoint rooted and labeled with metadata using iTOL v.5 (47).

### Gene-based comparisons.

Distributions of the predicted protein-coding genes of the 52 ST258 genomes were compared using Large Scale-BLAST Score Ratio (LS-BSR) analysis as previously described (48, 49). Protein-coding genes of each genome were predicted using Prodigal (50) and were grouped into clusters of related genes with ≥90% nucleotide identity and ≥90% alignment length using CD-HIT v.4.7 (51, 52). BLASTN (53, 54) was used to detect the gene clusters in each of the ST258 genomes analyzed, and the bit score was used to calculate a BSR value that demonstrates the level of similarity of each gene cluster in the genomes analyzed (48, 49). The annotation of each gene cluster was obtained using an in-house annotation pipeline (55). Statistically significant associations following multiple testing correction for each gene cluster with the different metadata categories (hospital, ICU, sample type, transmission, etc.) were determined using Scoary v.1.6.16 (56).

### Multilocus sequence typing (MLST).

A sequence type (ST) was determined for each CRKp genome using the seven-locus scheme developed by Diancourt et al. (57). Allele numbers and a sequence type were obtained for each CRKp isolate using the BIGSdb (58) software hosted by the Institut Pasteur (https://bigsdb.pasteur.fr/klebsiella/). Clonal groups (CGs) were designated for CRKp isolates that had closely related STs with the CG number representing the most prevalent ST or multiple STs within each group as consistent with previous studies (26–28).

### O antigen and K locus typing.

The O antigen types and K locus types were determined for each CRKp assembly using Kaptive v.0.5.1 (30, 59) and Kleborate v.2.2.0 (25). Only the matches predicted with a confidence of good or better are reported.

### Detection of mobile elements, antibiotic resistance genes, and virulence genes.

Plasmid types were identified using BLASTN (53, 54) to search the CRKp genomes for representative sequences from the PlasmidFinder database v.2021-11-29 (60, 61). A plasmid type was considered present in a genome when the representative sequences from the PlasmidFinder database were detected with the default parameters of ≥95% nucleotide identity and ≥60% alignment length. Predicted genes of the pKPC-CAV1193 (CP013325) plasmid were identified in each of the CRKp genomes by BLASTN BSR as previously described (62, 63).

Antibiotic resistance genes were identified in each genome using the resistance gene identifier (RGI) v.5.2.0 with the comprehensive antibiotic resistance database (CARD) v.3.1.4 (64). All perfect hits to previously described acquired antibiotic resistance genes were included while the strict hits were filtered to include only predictions that had ≥90% identity and ≥80% alignment length. The antibiotic resistance genes that are intrinsic among K. pneumoniae isolates, including chromosomally encoded efflux pumps and transcriptional regulators, as well as *fosA* and *bla*_SHV_ alleles, were excluded from our analysis of acquired resistance genes.

The presence or absence of K. pneumoniae virulence genes from the virulence factor database (VFDB) (65), was determined for each of the CRKp genomes analyzed using TBLASTN BSR (66) as previously described (48, 49, 67). Genes that were detected with a TBLASTN BSR value of ≥0.8 were considered present. The yersiniabactin sequence types (YbST) and colibactin sequence types (CbST) (28) were determined using the BIGSdb (58) software hosted by the Institut Pasteur (https://bigsdb.pasteur.fr/). The *ybt* and *cbt* lineages were determined using Kleborate v.2.2.0 (25).

Heat maps representing the prevalence and cooccurrence of virulence factors, acquired antibiotic resistance genes, or plasmid types among the CRKp genomes were generated with the ComplexHeatmap (68) package of R v.4.0.2 (69).

### Statistical analyses.

We estimated associations between HCP glove or gown contamination with CRKp and (i) health care personnel type and (ii) whether the HCP touched the patient or the environmental domain. Risk factors significant at an α value of ≤0.05 in the bivariate analyses were considered candidate predictors for the multivariable model. Models were built using logistic regression models fit by generalized estimating equations with an exchangeable correlation matrix to take into account within-patient correlation.

Statistical associations of individual virulence or antibiotic resistance genes, plasmid types, or lineages (ST or CG) with transmission, hospital, ICU versus non-ICU, clinical versus surveillance, and sample types (blood, wound, etc.) were determined using chi-square test or Fisher’s exact test where appropriate with R v.4.0.2 (69).

### Data availability.

All raw read files and assemblies generated for the CRKp isolates in this study are deposited in GenBank under BioProject accession no. PRJNA633565. Individual assembly accession numbers for each CRKp isolate are listed in Data Set S1.

## References

[1] Gupta N, Limbago BM, Patel JB, Kallen AJ. 2011. Carbapenem-resistant *Enterobacteriaceae*: epidemiology and prevention. Clin Infect Dis 53:60–67. doi:10.1093/cid/cir202. 21653305

[2] Centers for Disease Control and Prevention. 2019. Antibiotic resistance threats in the United States, 2019. Centers for Disease Control and Prevention, Atlanta, GA.

[3] World Health Organization. 2017. Global priority list of antibiotic-resistant bacteria to guide research, discovery, and development of new antibiotics. https://www.who.int/news/item/27-02-2017-who-publishes-list-of-bacteria-for-which-new-antibiotics-are-urgently-needed.

[4] Ramos-Castaneda JA, Ruano-Ravina A, Barbosa-Lorenzo R, Paillier-Gonzalez JE, Saldana-Campos JC, Salinas DF, Lemos-Luengas EV. 2018. Mortality due to KPC carbapenemase-producing Klebsiella pneumoniae infections: systematic review and meta-analysis: mortality due to KPC *Klebsiella pneumoniae* infections. J Infect 76:438–448. doi:10.1016/j.jinf.2018.02.007. 29477802

[5] Xu L, Sun X, Ma X. 2017. Systematic review and meta-analysis of mortality of patients infected with carbapenem-resistant *Klebsiella pneumoniae*. Ann Clin Microbiol Antimicrob 16:18. doi:10.1186/s12941-017-0191-3. 28356109 PMC5371217

[6] Paczosa MK, Mecsas J. 2016. *Klebsiella pneumoniae*: going on the offense with a strong defense. Microbiol Mol Biol Rev 80:629–661. doi:10.1128/MMBR.00078-15. 27307579 PMC4981674

[7] Pitout JD, Nordmann P, Poirel L. 2015. Carbapenemase-producing *Klebsiella pneumoniae*, a key pathogen set for global nosocomial dominance. Antimicrob Agents Chemother 59:5873–5884. doi:10.1128/AAC.01019-15. 26169401 PMC4576115

[8] Nordmann P, Cuzon G, Naas T. 2009. The real threat of *Klebsiella pneumoniae* carbapenemase-producing bacteria. Lancet Infect Dis 9:228–236. doi:10.1016/S1473-3099(09)70054-4. 19324295

[9] Chen L, Mathema B, Chavda KD, DeLeo FR, Bonomo RA, Kreiswirth BN. 2014. Carbapenemase-producing *Klebsiella pneumoniae*: molecular and genetic decoding. Trends Microbiol 22:686–696. doi:10.1016/j.tim.2014.09.003. 25304194 PMC4365952

[10] Chen L, Mathema B, Pitout JD, DeLeo FR, Kreiswirth BN. 2014. Epidemic *Klebsiella pneumoniae* ST258 is a hybrid strain. mBio 5:e01355-14. doi:10.1128/mBio.01355-14. 24961694 PMC4073492

[11] Deleo FR, Chen L, Porcella SF, Martens CA, Kobayashi SD, Porter AR, Chavda KD, Jacobs MR, Mathema B, Olsen RJ, Bonomo RA, Musser JM, Kreiswirth BN. 2014. Molecular dissection of the evolution of carbapenem-resistant multilocus sequence type 258 *Klebsiella pneumoniae*. Proc Natl Acad Sci USA 111:4988–4993. doi:10.1073/pnas.1321364111. 24639510 PMC3977278

[12] Kitchel B, Rasheed JK, Patel JB, Srinivasan A, Navon-Venezia S, Carmeli Y, Brolund A, Giske CG. 2009. Molecular epidemiology of KPC-producing *Klebsiella pneumoniae* isolates in the United States: clonal expansion of multilocus sequence type 258. Antimicrob Agents Chemother 53:3365–3370. doi:10.1128/AAC.00126-09. 19506063 PMC2715580

[13] Snitkin ES, Zelazny AM, Thomas PJ, Stock F, Henderson DK, Palmore TN, Segre JA, NISC Comparative Sequencing Program Group. 2012. Tracking a hospital outbreak of carbapenem-resistant *Klebsiella pneumoniae* with whole-genome sequencing. Sci Transl Med 4:148ra116. doi:10.1126/scitranslmed.3004129. PMC3521604 22914622

[14] Harris AD, Perencevich EN, Johnson JK, Paterson DL, Morris JG, Strauss SM, Johnson JA. 2007. Patient-to-patient transmission is important in extended-spectrum beta-lactamase-producing *Klebsiella pneumoniae* acquisition. Clin Infect Dis 45:1347–1350. doi:10.1086/522657. 17968833

[15] Harris AD, Pineles L, Belton B, Johnson JK, Shardell M, Loeb M, Newhouse R, Dembry L, Braun B, Perencevich EN, Hall KK, Morgan DJ, Benefits of Universal Glove and Gown (BUGG) Investigators, Shahryar SK, Price CS, Gadbaw JJ, Drees M, Kett DH, Munoz-Price LS, Jacob JT, Herwaldt LA, Sulis CA, Yokoe DS, Maragakis L, Lissauer ME, Zervos MJ, Warren DK, Carver RL, Anderson DJ, Calfee DP, Bowling JE, Safdar N. 2013. Universal glove and gown use and acquisition of antibiotic-resistant bacteria in the ICU: a randomized trial. JAMA 310:1571–1580. doi:10.1001/jama.2013.277815. 24097234 PMC4026208

[16] Snyder GM, Thom KA, Furuno JP, Perencevich EN, Roghmann MC, Strauss SM, Netzer G, Harris AD. 2008. Detection of methicillin-resistant *Staphylococcus aureus* and vancomycin-resistant enterococci on the gowns and gloves of healthcare workers. Infect Control Hosp Epidemiol 29:583–589. doi:10.1086/588701. 18549314 PMC2577846

[17] O’Hara LM, Calfee DP, Miller LG, Pineles L, Magder LS, Johnson JK, Morgan DJ, Harris AD. 2019. Optimizing contact precautions to curb the spread of antibiotic-resistant bacteria in hospitals: a multicenter cohort study to identify patient characteristics and healthcare personnel interactions associated with transmission of methicillin-resistant *Staphylococcus aureus*. Clin Infect Dis 69:S171–S177. doi:10.1093/cid/ciz621. 31517979 PMC6761365

[18] O’Hara LM, Nguyen MH, Calfee DP, Miller LG, Pineles L, Magder LS, Johnson JK, Morgan DJ, Rasko DA, Harris AD, CDC Prevention Epicenters Program. 2021. Risk factors for transmission of carbapenem-resistant *Enterobacterales* to healthcare personnel gloves and gowns in the USA. J Hosp Infect 109:58–64. doi:10.1016/j.jhin.2020.12.012. 33358930 PMC8211026

[19] Rock C, Thom KA, Masnick M, Johnson JK, Harris AD, Morgan DJ. 2014. Frequency of *Klebsiella pneumoniae* carbapenemase (KPC)-producing and non-KPC-producing *Klebsiella* species contamination of healthcare workers and the environment. Infect Control Hosp Epidemiol 35:426–429. doi:10.1086/675598. 24602950 PMC4030386

[20] Long SW, Olsen RJ, Eagar TN, Beres SB, Zhao P, Davis JJ, Brettin T, Xia F, Musser JM. 2017. Population genomic analysis of 1,777 extended-spectrum beta-lactamase-producing *Klebsiella pneumoniae* isolates, Houston, Texas: unexpected abundance of clonal group 307. mBio 8:e00489-17. doi:10.1128/mBio.00489-17. 28512093 PMC5433097

[21] Wyres KL, Wick RR, Judd LM, Froumine R, Tokolyi A, Gorrie CL, Lam MMC, Duchene S, Jenney A, Holt KE. 2019. Distinct evolutionary dynamics of horizontal gene transfer in drug resistant and virulent clones of *Klebsiella pneumoniae*. PLoS Genet 15:e1008114. doi:10.1371/journal.pgen.1008114. 30986243 PMC6483277

[22] Marsh JW, Mustapha MM, Griffith MP, Evans DR, Ezeonwuka C, Pasculle AW, Shutt KA, Sundermann A, Ayres AM, Shields RK, Babiker A, Cooper VS, Van Tyne D, Harrison LH. 2019. Evolution of outbreak-causing carbapenem-resistant *Klebsiella pneumoniae* ST258 at a tertiary care hospital over 8 Years. mBio 10:e01945-19. doi:10.1128/mBio.01945-19. 31481386 PMC6722418

[23] Holt KE, Wertheim H, Zadoks RN, Baker S, Whitehouse CA, Dance D, Jenney A, Connor TR, Hsu LY, Severin J, Brisse S, Cao H, Wilksch J, Gorrie C, Schultz MB, Edwards DJ, Nguyen KV, Nguyen TV, Dao TT, Mensink M, Minh VL, Nhu NT, Schultsz C, Kuntaman K, Newton PN, Moore CE, Strugnell RA, Thomson NR. 2015. Genomic analysis of diversity, population structure, virulence, and antimicrobial resistance in *Klebsiella pneumoniae*, an urgent threat to public health. Proc Natl Acad Sci USA 112:E3574–E3581. doi:10.1073/pnas.1501049112. 26100894 PMC4500264

[24] Adediran T, Harris AD, Johnson J, Calfee DP, Miller LG, Nguyen MH, Morgan DJ, Goodman KE, Hitchcock S, Pineles L, O’Hara LM. 2020. Epidemiologic and microbiologic characteristics of hospitalized patients co-colonized with multiple species of carbapenem-resistant *Enterobacteriaceae* in the United States. Open Forum Infect Dis 7:ofaa386. doi:10.1093/ofid/ofaa386. 33072811 PMC7539689

[25] Lam MMC, Wick RR, Watts SC, Cerdeira LT, Wyres KL, Holt KE. 2021. A genomic surveillance framework and genotyping tool for *Klebsiella pneumoniae* and its related species complex. Nat Commun 12:4188. doi:10.1038/s41467-021-24448-3. 34234121 PMC8263825

[26] Bialek-Davenet S, Criscuolo A, Ailloud F, Passet V, Jones L, Delannoy-Vieillard AS, Garin B, Le Hello S, Arlet G, Nicolas-Chanoine MH, Decre D, Brisse S. 2014. Genomic definition of hypervirulent and multidrug-resistant *Klebsiella pneumoniae* clonal groups. Emerg Infect Dis 20:1812–1820. doi:10.3201/eid2011.140206. 25341126 PMC4214299

[27] Wyres KL, Lam MMC, Holt KE. 2020. Population genomics of *Klebsiella pneumoniae*. Nat Rev Microbiol 18:344–359. doi:10.1038/s41579-019-0315-1. 32055025

[28] Lam MMC, Wick RR, Wyres KL, Gorrie CL, Judd LM, Jenney AWJ, Brisse S, Holt KE. 2018. Genetic diversity, mobilisation and spread of the yersiniabactin-encoding mobile element ICEKp in *Klebsiella pneumoniae* populations. Microb Genom 4:e000196. doi:10.1099/mgen.0.000196. 29985125 PMC6202445

[29] Pennini ME, De Marco A, Pelletier M, Bonnell J, Cvitkovic R, Beltramello M, Cameroni E, Bianchi S, Zatta F, Zhao W, Xiao X, Camara MM, DiGiandomenico A, Semenova E, Lanzavecchia A, Warrener P, Suzich J, Wang Q, Corti D, Stover CK. 2017. Immune stealth-driven O2 serotype prevalence and potential for therapeutic antibodies against multidrug resistant *Klebsiella pneumoniae*. Nat Commun 8:1991. doi:10.1038/s41467-017-02223-7. 29222409 PMC5722860

[30] Wick RR, Heinz E, Holt KE, Wyres KL. 2018. Kaptive web: user-friendly capsule and lipopolysaccharide serotype prediction for *Klebsiella* genomes. J Clin Microbiol 56:e00197-18. doi:10.1128/JCM.00197-18. 29618504 PMC5971559

[31] Fajardo-Lubian A, Ben Zakour NL, Agyekum A, Qi Q, Iredell JR. 2019. Host adaptation and convergent evolution increases antibiotic resistance without loss of virulence in a major human pathogen. PLoS Pathog 15:e1007218. doi:10.1371/journal.ppat.1007218. 30875398 PMC6436753

[32] Potron A, Rondinaud E, Poirel L, Belmonte O, Boyer S, Camiade S, Nordmann P. 2013. Genetic and biochemical characterisation of OXA-232, a carbapenem-hydrolysing class D beta-lactamase from Enterobacteriaceae. Int J Antimicrob Agents 41:325–329. doi:10.1016/j.ijantimicag.2012.11.007. 23305656

[33] Hamzaoui Z, Ocampo-Sosa A, Fernandez Martinez M, Landolsi S, Ferjani S, Maamar E, Saidani M, Slim A, Martinez-Martinez L, Boutiba-Ben Boubaker I. 2018. Role of association of OmpK35 and OmpK36 alteration and blaESBL and/or blaAmpC genes in conferring carbapenem resistance among non-carbapenemase-producing *Klebsiella pneumoniae*. Int J Antimicrob Agents 52:898–905. doi:10.1016/j.ijantimicag.2018.03.020. 29621592

[34] Liakopoulos A, Mevius D, Ceccarelli D. 2016. A review of SHV extended-spectrum beta-lactamases: neglected yet ubiquitous. Front Microbiol 7:1374. doi:10.3389/fmicb.2016.01374. 27656166 PMC5011133

[35] Sheppard AE, Stoesser N, Sebra R, Kasarskis A, Deikus G, Anson L, Walker AS, Peto TE, Crook DW, Mathers AJ. 2016. Complete genome sequence of KPC-producing *Klebsiella pneumoniae* strain CAV1193. Genome Announc 4:e01649-15. doi:10.1128/genomeA.01649-15. 26823590 PMC4732343

[36] Villa L, Feudi C, Fortini D, Brisse S, Passet V, Bonura C, Endimiani A, Mammina C, Ocampo AM, Jimenez JN, Doumith M, Woodford N, Hopkins K, Carattoli A. 2017. Diversity, virulence, and antimicrobial resistance of the KPC-producing *Klebsiella pneumoniae* ST307 clone. Microb Genom 3:e000110. doi:10.1099/mgen.0.000110. 28785421 PMC5506382

[37] Lowe M, Kock MM, Coetzee J, Hoosien E, Peirano G, Strydom KA, Ehlers MM, Mbelle NM, Shashkina E, Haslam DB, Dhawan P, Donnelly RJ, Chen L, Kreiswirth BN, Pitout JDD. 2019. *Klebsiella pneumoniae* ST307 with blaOXA-181, South Africa, 2014–2016. Emerg Infect Dis 25:739–747. doi:10.3201/eid2504.181482. 30882333 PMC6433043

[38] Wyres KL, Hawkey J, Hetland MAK, Fostervold A, Wick RR, Judd LM, Hamidian M, Howden BP, Lohr IH, Holt KE. 2019. Emergence and rapid global dissemination of CTX-M-15-associated *Klebsiella pneumoniae* strain ST307. J Antimicrob Chemother 74:577–581. doi:10.1093/jac/dky492. 30517666 PMC6376852

[39] Lapp Z, Han JH, Wiens J, Goldstein EJC, Lautenbach E, Snitkin ES. 2021. Patient and microbial genomic factors associated with carbapenem-resistant *Klebsiella pneumoniae* extraintestinal colonization and infection. mSystems 6:e00177-21. doi:10.1128/mSystems.00177-21. 33727393 PMC8546970

[40] Clinical and Laboratory Standards Institute. 2020. Performance standards for antimicrobial susceptibility testing, 30th ed. Clinical and Laboratory Standards Institute, Wayne, PA.

[41] Bolger AM, Lohse M, Usadel B. 2014. Trimmomatic: a flexible trimmer for Illumina sequence data. Bioinformatics 30:2114–2120. doi:10.1093/bioinformatics/btu170. 24695404 PMC4103590

[42] Nurk S, Bankevich A, Antipov D, Gurevich AA, Korobeynikov A, Lapidus A, Prjibelski AD, Pyshkin A, Sirotkin A, Sirotkin Y, Stepanauskas R, Clingenpeel SR, Woyke T, McLean JS, Lasken R, Tesler G, Alekseyev MA, Pevzner PA. 2013. Assembling single-cell genomes and mini-metagenomes from chimeric MDA products. J Comput Biol 20:714–737. doi:10.1089/cmb.2013.0084. 24093227 PMC3791033

[43] Chaumeil PA, Mussig AJ, Hugenholtz P, Parks DH. 2022. GTDB-Tk v2: memory friendly classification with the genome taxonomy database. Bioinformatics 38:5315–5316. doi:10.1093/bioinformatics/btac672. 36218463 PMC9710552

[44] Sahl JW, Lemmer D, Travis J, Schupp JM, Gillece JD, Aziz M, Driebe EM, Drees KP, Hicks ND, Williamson CHD, Hepp CM, Smith DE, Roe C, Engelthaler DM, Wagner DM, Keim P. 2016. NASP: an accurate, rapid method for the identification of SNPs in WGS datasets that supports flexible input and output formats. Microb Genom 2:e000074. doi:10.1099/mgen.0.000074. 28348869 PMC5320593

[45] Nguyen LT, Schmidt HA, von Haeseler A, Minh BQ. 2015. IQ-TREE: a fast and effective stochastic algorithm for estimating maximum-likelihood phylogenies. Mol Biol Evol 32:268–274. doi:10.1093/molbev/msu300. 25371430 PMC4271533

[46] Hoang DT, Chernomor O, von Haeseler A, Minh BQ, Vinh LS. 2018. UFBoot2: improving the ultrafast bootstrap approximation. Mol Biol Evol 35:518–522. doi:10.1093/molbev/msx281. 29077904 PMC5850222

[47] Letunic I, Bork P. 2019. Interactive Tree Of Life (iTOL) v4: recent updates and new developments. Nucleic Acids Res 47:W256–W259. doi:10.1093/nar/gkz239. 30931475 PMC6602468

[48] Sahl JW, Caporaso JG, Rasko DA, Keim P. 2014. The large-scale blast score ratio (LS-BSR) pipeline: a method to rapidly compare genetic content between bacterial genomes. PeerJ 2:e332. doi:10.7717/peerj.332. 24749011 PMC3976120

[49] Hazen TH, Donnenberg MS, Panchalingam S, Antonio M, Hossain A, Mandomando I, Ochieng JB, Ramamurthy T, Tamboura B, Qureshi S, Quadri F, Zaidi A, Kotloff KL, Levine MM, Barry EM, Kaper JB, Rasko DA, Nataro JP. 2016. Genomic diversity of EPEC associated with clinical presentations of differing severity. Nat Microbiol 1:15014. doi:10.1038/nmicrobiol.2015.14. 27571975 PMC5067155

[50] Hyatt D, Chen GL, Locascio PF, Land ML, Larimer FW, Hauser LJ. 2010. Prodigal: prokaryotic gene recognition and translation initiation site identification. BMC Bioinformatics 11:119. doi:10.1186/1471-2105-11-119. 20211023 PMC2848648

[51] Fu L, Niu B, Zhu Z, Wu S, Li W. 2012. CD-HIT: accelerated for clustering the next-generation sequencing data. Bioinformatics 28:3150–3152. doi:10.1093/bioinformatics/bts565. 23060610 PMC3516142

[52] Li W, Godzik A. 2006. Cd-hit: a fast program for clustering and comparing large sets of protein or nucleotide sequences. Bioinformatics 22:1658–1659. doi:10.1093/bioinformatics/btl158. 16731699

[53] Camacho C, Coulouris G, Avagyan V, Ma N, Papadopoulos J, Bealer K, Madden TL. 2009. BLAST+: architecture and applications. BMC Bioinformatics 10:421. doi:10.1186/1471-2105-10-421. 20003500 PMC2803857

[54] Altschul SF, Gish W, Miller W, Myers EW, Lipman DJ. 1990. Basic local alignment search tool. J Mol Biol 215:403–410. doi:10.1016/S0022-2836(05)80360-2. 2231712

[55] Galens K, Orvis J, Daugherty S, Creasy HH, Angiuoli S, White O, Wortman J, Mahurkar A, Giglio MG. 2011. The IGS standard operating procedure for automated prokaryotic annotation. Stand Genomic Sci 4:244–251. doi:10.4056/sigs.1223234. 21677861 PMC3111993

[56] Brynildsrud O, Bohlin J, Scheffer L, Eldholm V. 2016. Rapid scoring of genes in microbial pan-genome-wide association studies with Scoary. Genome Biol 17:238. doi:10.1186/s13059-016-1108-8. 27887642 PMC5124306

[57] Diancourt L, Passet V, Verhoef J, Grimont PA, Brisse S. 2005. Multilocus sequence typing of *Klebsiella pneumoniae* nosocomial isolates. J Clin Microbiol 43:4178–4182. doi:10.1128/JCM.43.8.4178-4182.2005. 16081970 PMC1233940

[58] Jolley KA, Maiden MC. 2010. BIGSdb: scalable analysis of bacterial genome variation at the population level. BMC Bioinformatics 11:595. doi:10.1186/1471-2105-11-595. 21143983 PMC3004885

[59] Wyres KL, Wick RR, Gorrie C, Jenney A, Follador R, Thomson NR, Holt KE. 2016. Identification of *Klebsiella* capsule synthesis loci from whole genome data. Microb Genom 2:e000102. doi:10.1099/mgen.0.000102. 28348840 PMC5359410

[60] Carattoli A, Zankari E, Garcia-Fernandez A, Voldby Larsen M, Lund O, Villa L, Moller Aarestrup F, Hasman H. 2014. *In silico* detection and typing of plasmids using PlasmidFinder and plasmid multilocus sequence typing. Antimicrob Agents Chemother 58:3895–3903. doi:10.1128/AAC.02412-14. 24777092 PMC4068535

[61] Carattoli A, Hasman H. 2020. PlasmidFinder and in silico pMLST: identification and typing of plasmid replicons in whole-genome sequencing (WGS). Methods Mol Biol 2075:285–294. doi:10.1007/978-1-4939-9877-7_20. 31584170

[62] Hazen TH, Mettus R, McElheny CL, Bowler SL, Nagaraj S, Doi Y, Rasko DA. 2018. Diversity among *bla*KPC-containing plasmids in *Escherichia coli* and other bacterial species isolated from the same patients. Sci Rep 8:10291. doi:10.1038/s41598-018-28085-7. 29980699 PMC6035167

[63] Hazen TH, Michalski J, Nagaraj S, Okeke IN, Rasko DA. 2017. Characterization of a large antibiotic resistance plasmid found in enteropathogenic *Escherichia coli* strain B171 and its relatedness to plasmids of diverse *E. coli* and *Shigella* strains. Antimicrob Agents Chemother 61:e00995-17. doi:10.1128/AAC.00995-17. 28674052 PMC5571317

[64] Alcock BP, Raphenya AR, Lau TTY, Tsang KK, Bouchard M, Edalatmand A, Huynh W, Nguyen AV, Cheng AA, Liu S, Min SY, Miroshnichenko A, Tran HK, Werfalli RE, Nasir JA, Oloni M, Speicher DJ, Florescu A, Singh B, Faltyn M, Hernandez-Koutoucheva A, Sharma AN, Bordeleau E, Pawlowski AC, Zubyk HL, Dooley D, Griffiths E, Maguire F, Winsor GL, Beiko RG, Brinkman FSL, Hsiao WWL, Domselaar GV, McArthur AG. 2020. CARD 2020: antibiotic resistome surveillance with the comprehensive antibiotic resistance database. Nucleic Acids Res 48:D517–D525. doi:10.1093/nar/gkz935. 31665441 PMC7145624

[65] Liu B, Zheng D, Jin Q, Chen L, Yang J. 2019. VFDB 2019: a comparative pathogenomic platform with an interactive web interface. Nucleic Acids Res 47:D687–D692. doi:10.1093/nar/gky1080. 30395255 PMC6324032

[66] Rasko DA, Myers GS, Ravel J. 2005. Visualization of comparative genomic analyses by BLAST score ratio. BMC Bioinformatics 6:2. doi:10.1186/1471-2105-6-2. 15634352 PMC545078

[67] Hazen TH, Sahl JW, Fraser CM, Donnenberg MS, Scheutz F, Rasko DA. 2013. Refining the pathovar paradigm via phylogenomics of the attaching and effacing *Escherichia coli*. Proc Natl Acad Sci USA 110:12810–12815. doi:10.1073/pnas.1306836110. 23858472 PMC3732946

[68] Gu Z, Eils R, Schlesner M. 2016. Complex heatmaps reveal patterns and correlations in multidimensional genomic data. Bioinformatics 32:2847–2849. doi:10.1093/bioinformatics/btw313. 27207943

[69] R Core Team. 2020. R: a language and environment for statistical computing. R Foundation for Statistical Computing, Vienna, Austria. https://www.R-project.org/.

