# Supplementary material for: Clinical and Bacterial Characteristics Associated with Glove and Gown Contamination by Carbapenem-Resistant Klebsiella pneumoniae in the Health Care Setting
Source: Microbiol Spectr. 2023 Jun 8;11(4):e01775-23. doi: 10.1128/spectrum.01775-23 (PMC10434059; doi:10.1128/spectrum.01775-23)
Supplement: Supplemental file 3 — Fig. S1 to S5 and Table S1. Download spectrum.01775-23-s0001.pdf, PDF file, 2.8 MB [file spectrum.01775-23-s0001.pdf]

Supplemental Material for:

Clinical and bacterial characteristics associated with glove and gown contamination of carbapenem-resistant *Klebsiella pneumoniae* in the healthcare setting

Tracy H. Hazen[a,b]*, Timileyin Adediran[c], Stephanie Hitchcock[c], Lyndsay M. O'Hara[c], Lisa Pineles[c], Jane M. Michalski[a,b], J. Kristie Johnson[c], M. Hong Nguyen[d], David P. Calfee[e], Loren G. Miller[f], Anthony D. Harris[c], and David A. Rasko[a,b]

[a] Institute for Genome Sciences, [b] Department of Microbiology and Immunology,
[c] Department of Epidemiology and Public Health, University of Maryland School of Medicine, Baltimore, MD USA
[d] Department of Medicine, University of Pittsburgh, Pittsburgh, PA USA
[e] Division of Infectious Diseases, Weill Cornell Medicine, New York, NY USA
[f] Lundquist Institute at Harbor-UCLA Medical Center, Torrance, CA USA

Running title: CRKp contamination

*Corresponding author:
Tracy H. Hazen, Ph.D.
University of Maryland, School of Medicine
Institute for Genome Sciences
Department of Microbiology and Immunology
670 W. Baltimore Street, Room 2103
Baltimore, Maryland, USA 21201
e-mail: thazen@som.umaryland.edu
Phone: 410-706-1954
Fax: 410-706-1842

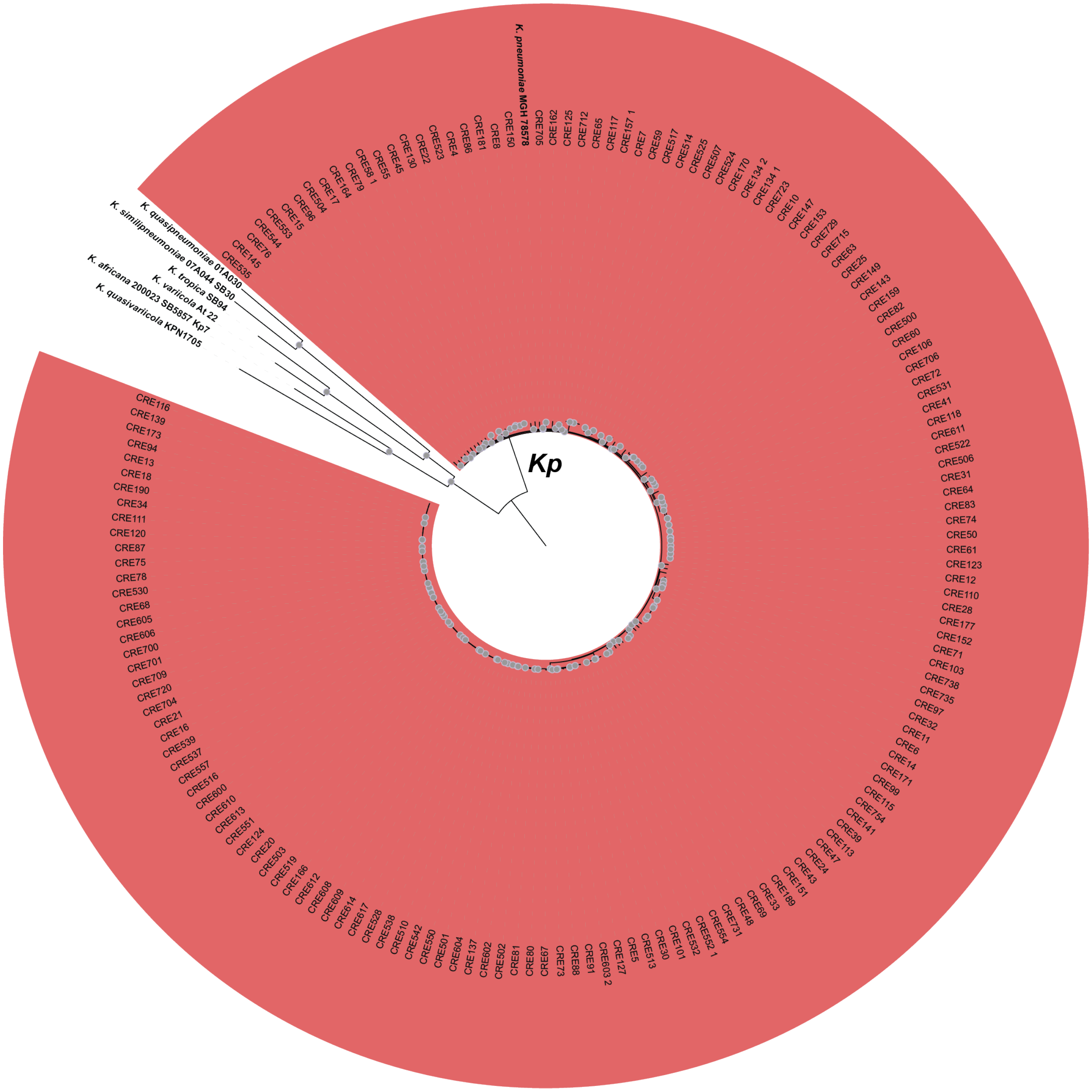

Figure S1. Phylogenomic analysis of the 166 CRKp analyzed in this study and representative genomes of the *K. pneumoniae* Species Complex (KpSC). The KpSC reference genomes include the species name in the label. Red indicates the *K. pneumoniae* clade. UFBoot values ≥95 are indicated by a gray circle.

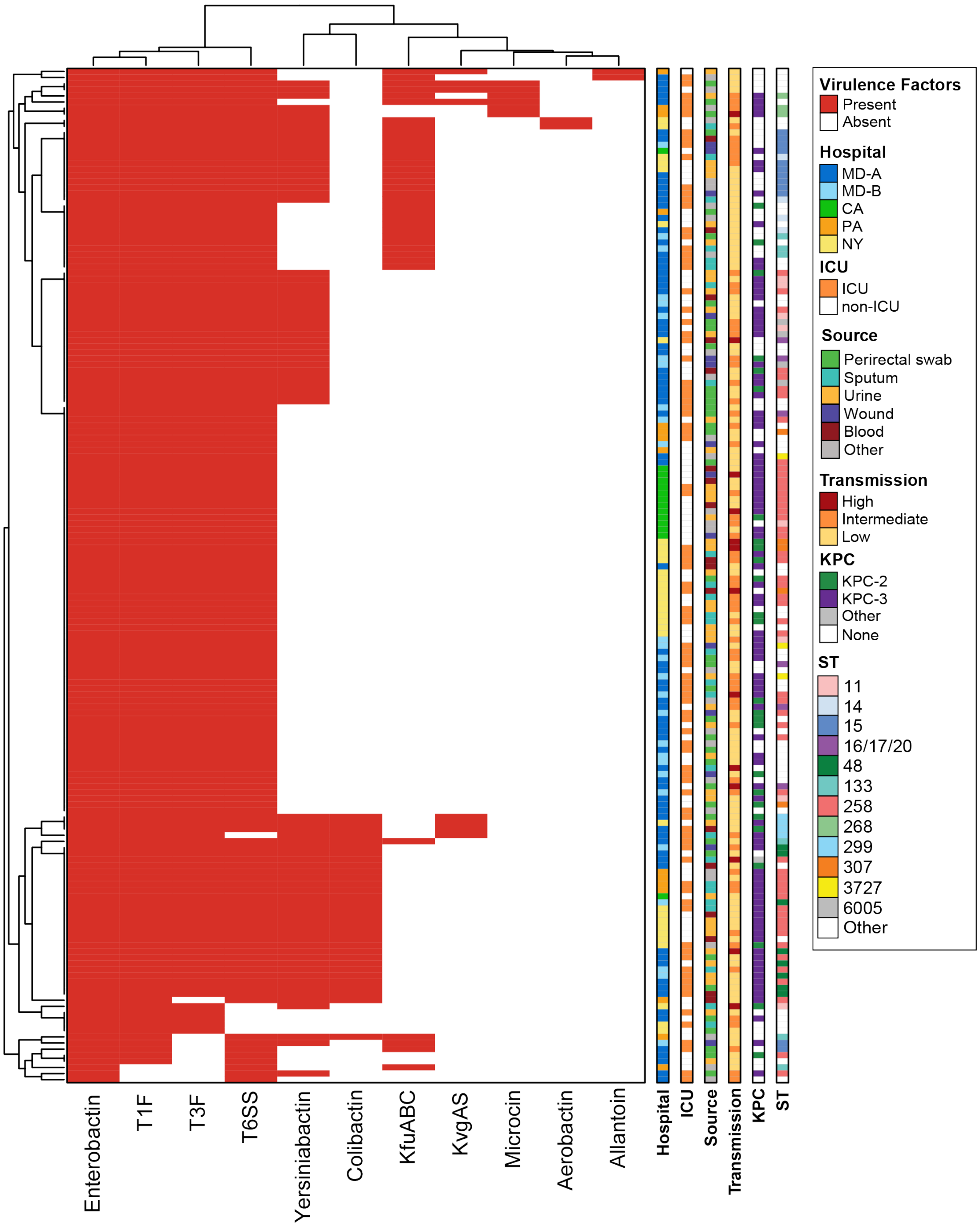

Figure S2. Distribution of known *K. pneumoniae* virulence factors among the CRKp. The heat map indicates the prevalence and co-occurrence of *K. pneumoniae* virulence genes (columns) among the 166 CRKp isolates (rows). Virulence factors identified in each genome are indicated by red while white indicates they were not detected. Metadata categories are to the right of the heat map.

A

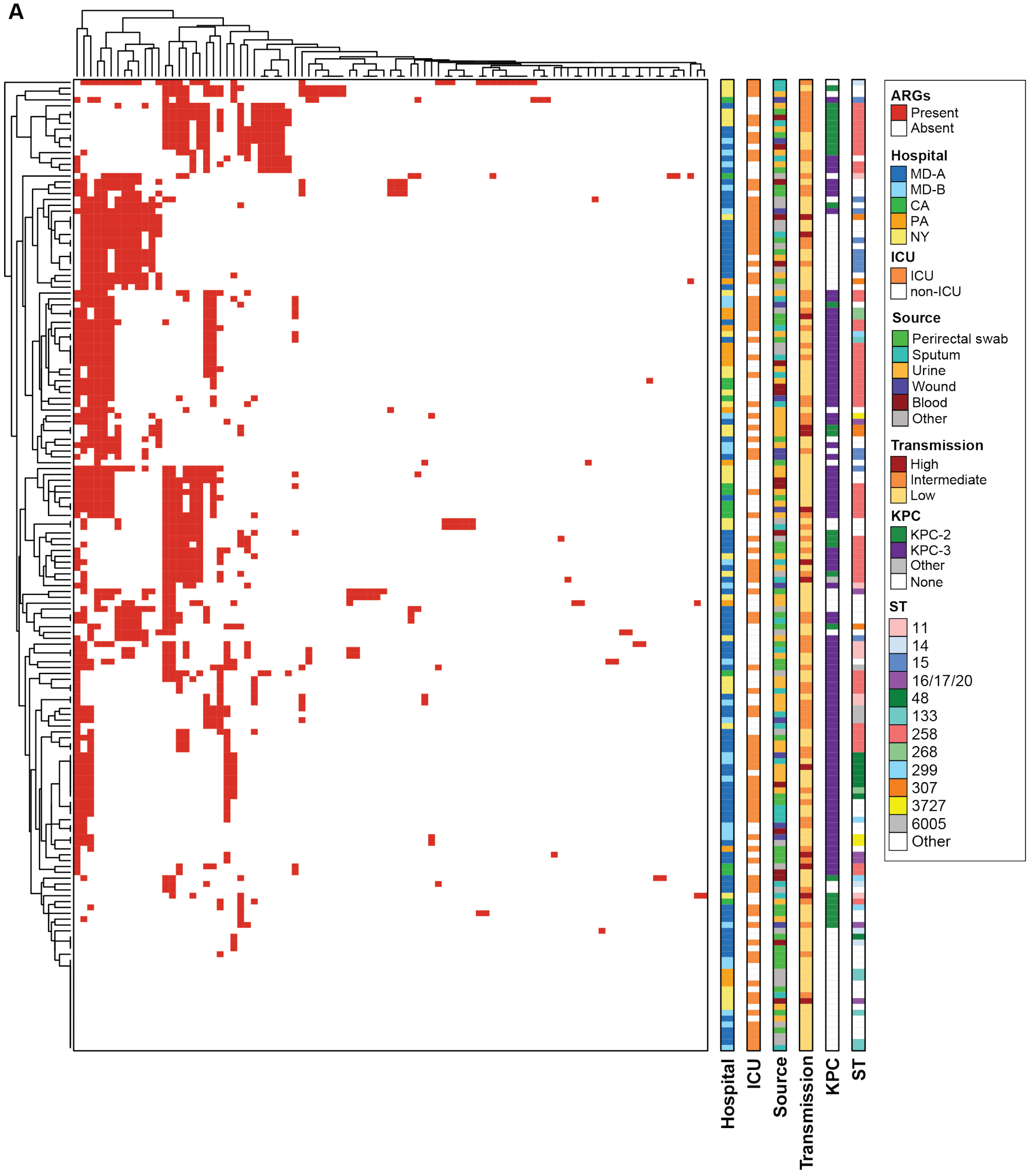
ARGs
Present
Absent
Hospital
MD-A
MD-B
CA
PA
NY
ICU
ICU
non-ICU
Source
Perirectal swab
Sputum
Urine
Wound
Blood
Other
Transmission
High
Intermediate
Low
KPC
KPC-2
KPC-3
Other
None
ST
11
14
15
16/17/20
48
133
258
268
299
307
3727
6005
Other
Hospital
ICU
Source
Transmission
KPC
ST

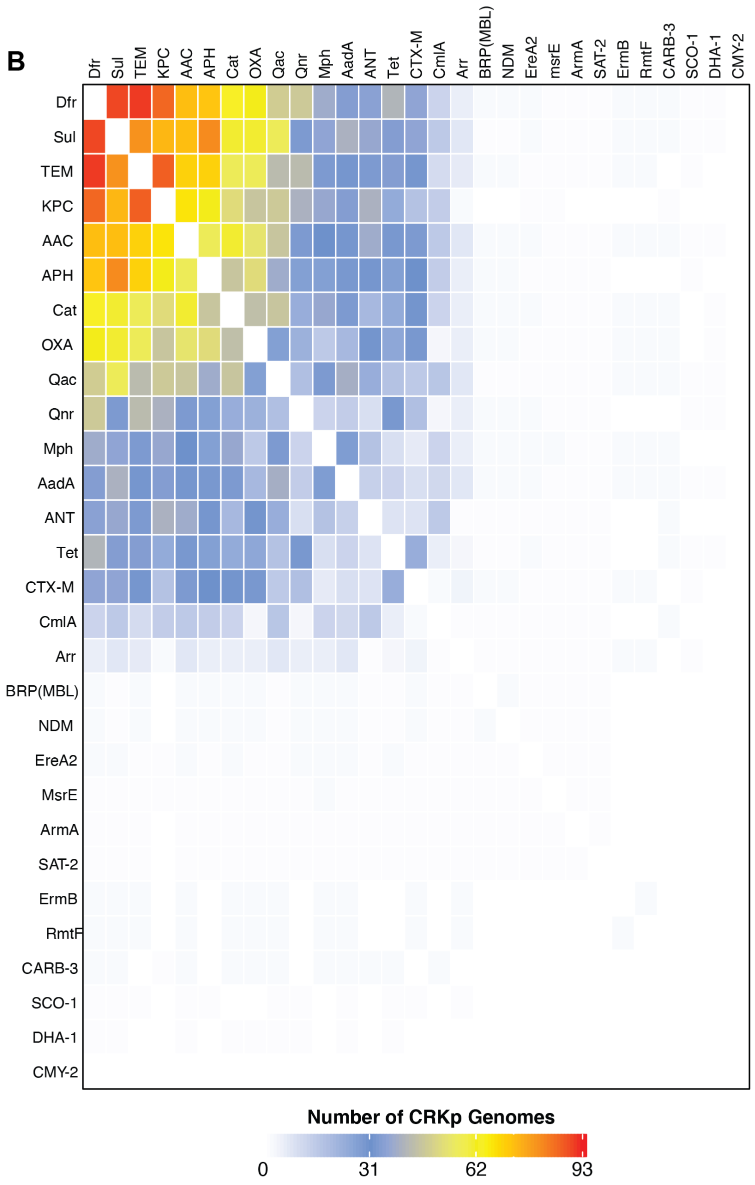

Figure S3. Prevalence and co-occurrence of antibiotic resistance genes (ARGs) among the CRKp. A) The heat map indicates the presence of acquired ARGs (columns) among the 166 CRKp isolates (rows). ARGs identified in each genome are indicated by red while white indicates they were not detected. Metadata categories are to the right of the heat map. **B)** Numbers of CRKp genomes that contain each ARG family demonstrating the most prevalent (red) to least prevalent (white) genes. ARGs identified at similar frequencies such as *dfr, sul,* and *bla*$_{\mathrm{TEM}}$ genes can co-occur on mobile elements.

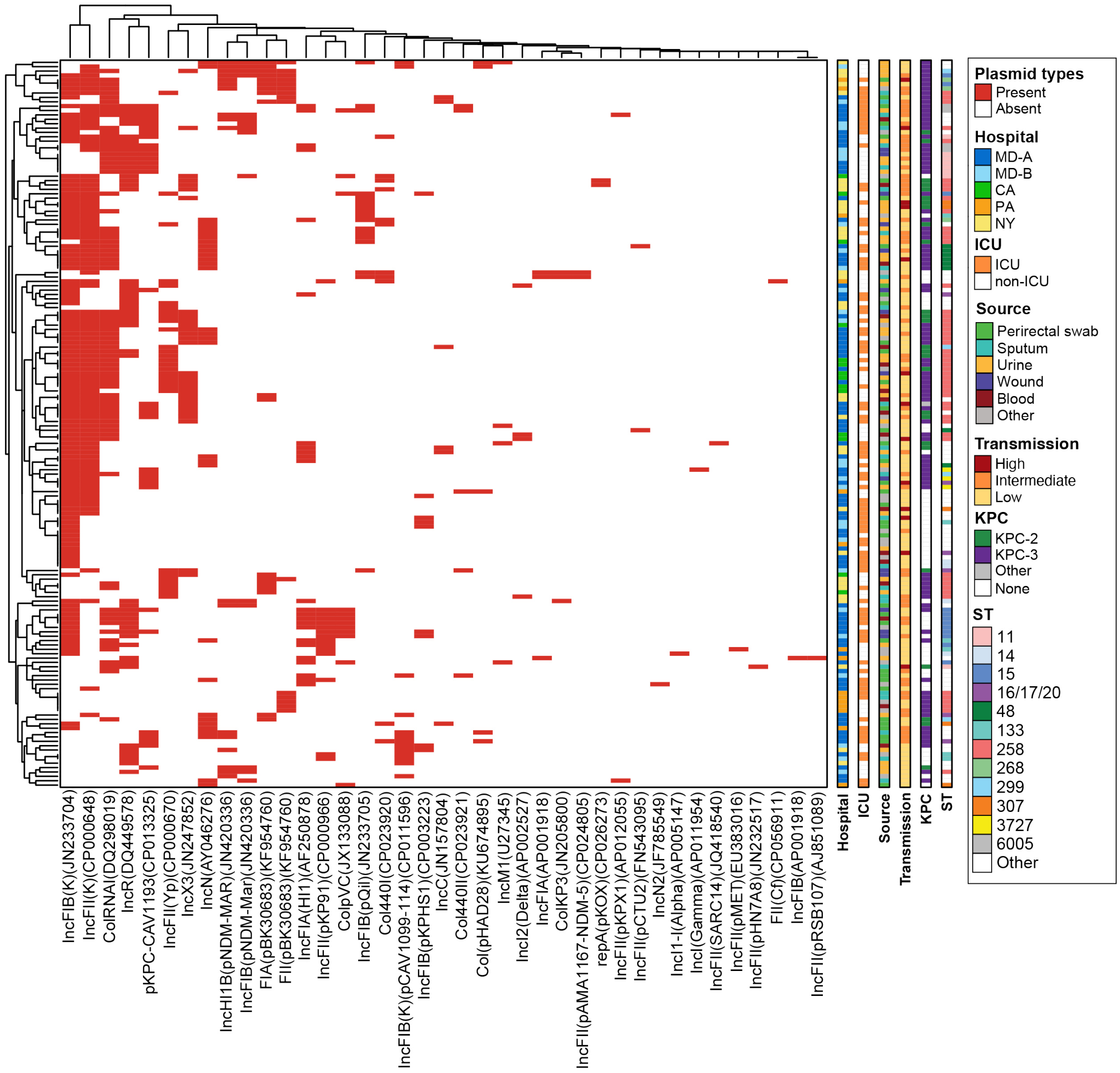

Figure S4. Prevalence of characterized plasmid types among the CRKp. The heat map indicates the prevalence and co-occurrence of previously described plasmids represented by their incompatibility (Inc) types (columns) among each of the 166 CRKp isolates (rows). Plasmid types identified in each genome are indicated by red while white indicates they were not detected. Metadata categories of the CRKp isolates are to the right of the heat map.

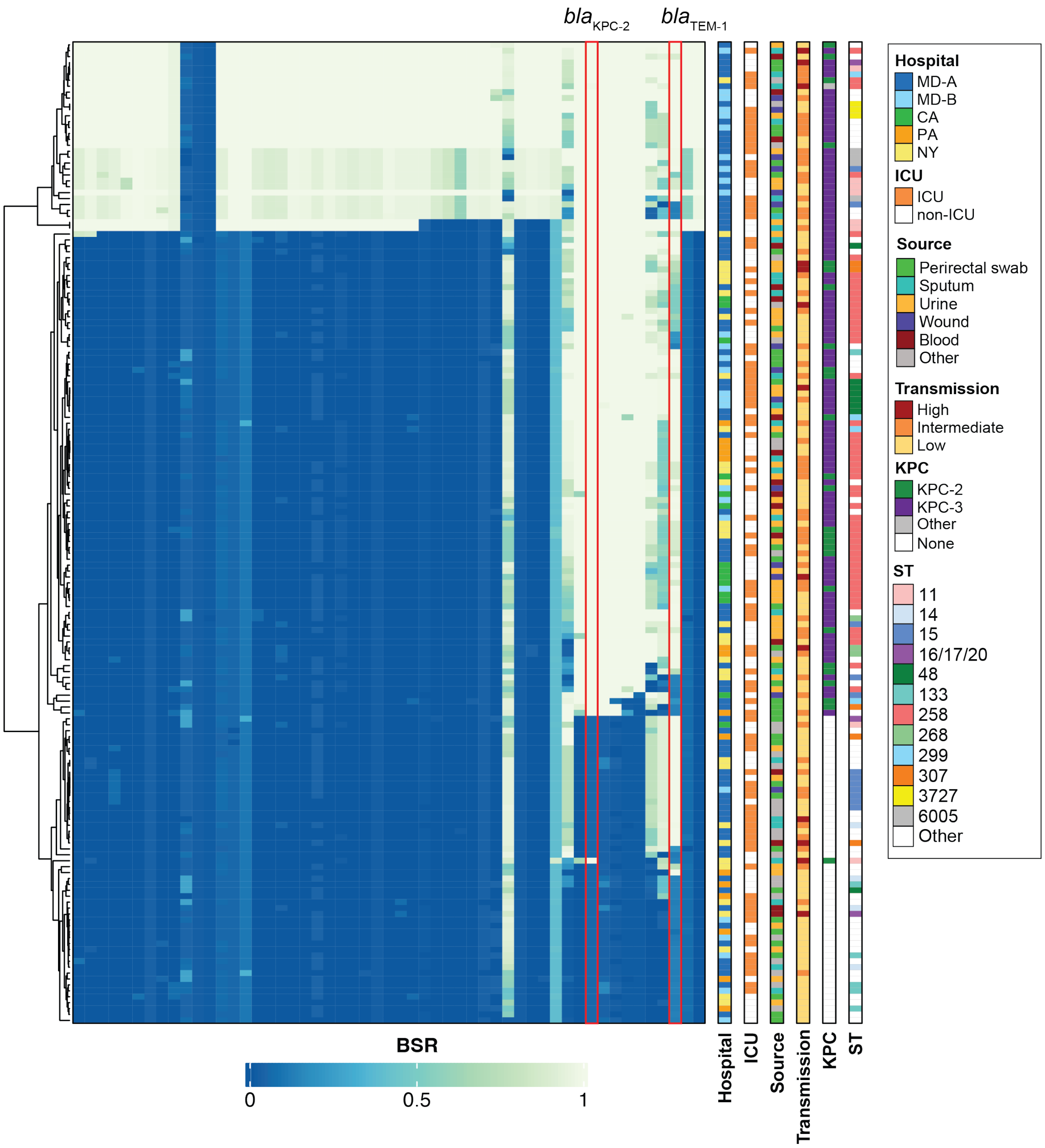

Figure S5. Distribution of plasmid pKPC-CAV1193 among the CRKp. The heat map indicates the presence of genes of plasmid pKPC-CAV1193 (CP013325) among the 166 CRKp isolates analyzed in this study. A BSR value was calculated for each gene against all the CRKp genomes, which estimates the level of similarity at which the genes were detected in each genome. Each column is a different gene of plasmid pKPC-CAV1193 while each row is a different CRKp genome. The *bla*$_{KPC-2}$ and *bla*$_{TEM-1}$ genes of pKPC-CAV1193 are indicated with a red box. Metadata categories of the CRKp isolates are to the right of the heat map.

Table S1. Association between touching patient and environmental domains and contamination of gloves or gown with CRKp

Domain touched (N=1,658)	Number of observations n (%)	Interactions resulting in contamination (%)	aOR (95% CI)	p-value
Patient only	286 (17.3)	12.2	1.07 (0.67-1.72)	0.78
Environment only	222 (13.4)	5.9	0.53 (0.28-1.00)	0.05
Any patient contact	1107 (66.8)	13.2	1.36 (0.90-2.08)	0.15
Nothing	43 (2.6)	2.3	0.19 (0.03-1.34)	0.09

Any patient contact: patient only or both patient and environment

Adjusted for: culture source, ICU status, and healthcare personnel type
